# Adaptive quadtree-based segmentation of nucleus and cytoplasm in pap-smear images: a lightweight and interpretable approach for automated cytology

**DOI:** 10.3389/frai.2026.1834763

**Published:** 2026-06-12

**Authors:** Wasswa William, Andrew Ware

**Affiliations:** 1 Department of Biomedical Sciences and Engineering, Mbarara University of Science and Technology, Mbarara, Uganda; 2 Faculty of Computing, Engineering and Science, University of South Wales, Pontypridd, United Kingdom

**Keywords:** cervical cytology, computer-aided diagnosis, cytoplasm segmentation, medical image analysis, nucleus segmentation, pap-smear, quadtree decomposition

## Abstract

**Background:**

Automated analysis of Pap-smear images plays an important role in cervical cancer screening, particularly in low-resource settings where manual cytology remains labour-intensive, subjective, and prone to inter-observer variability. On the other hand, accurate segmentation of the nucleus and cytoplasm is a fundamental step in computer-aided diagnosis systems because it enables quantitative morphometric analysis and computation of clinically important biomarkers such as the nucleus-to-cytoplasm ratio. However, robust cervical cell segmentation remains challenging due to staining variability, inhomogeneity, irregular morphology, weak cytoplasmic boundaries, and overlapping cellular structures. This study presents an adaptive quadtree-based segmentation framework for automated nucleus and cytoplasm delineation in Pap-smear images.

**Methods:**

The proposed method employs hierarchical split–merge decomposition guided by a dynamic adaptive statistical homogeneity analysis using mean intensity, variance, and entropy measures. Preprocessing is performed using large-kernel median filtering for background normalisation, followed by local Otsu thresholding, adaptive region merging, overlap refinement, and morphological post-processing. The framework was evaluated on both the Herlev cervical cytology dataset and the ISBI 2015 cervical cytology segmentation challenge dataset containing overlapping and clustered cervical cells. Comparative benchmarking was additionally performed against the U-Net and Attention U-Net.

**Results:**

On the Herlev dataset, the proposed framework achieved nucleus Dice coefficients exceeding 0.94 and Zijdenbos Similarity Index (ZSI) values greater than 0.9034 across all diagnostic classes, with competitive cytoplasm segmentation performance. On the ISBI 2015 dataset, the framework maintained acceptable segmentation performance under overlapping-cell conditions, achieving nucleus Dice and ZSI values of 0.912 ± 0.048 and 0.918 ± 0.044, respectively. Morphometric feature comparisons demonstrated strong agreement with ground-truth annotations and low average percentage errors for area and diameter measurements. Although deep learning models achieved superior performance under highly complex overlap conditions, the proposed framework remained competitive while requiring substantially lower computational resources and no iterative model training.

**Conclusion:**

The proposed Adaptive Quadtree-Based Segmentation framework provides a lightweight, interpretable, and computationally efficient approach for automated cervical cytology segmentation. Its training-free design, transparent statistical decision rules, and reduced hardware requirements make it particularly suitable for deployment in resource-constrained and embedded cervical cancer screening systems. The framework provides a practical segmentation backbone for automated cytology analysis and downstream computer-aided diagnosis applications.

## 1 Background

Cervical cancer remains one of the leading causes of cancer-related morbidity and mortality among women globally, with nearly 85% of the disease burden occurring in low- and middle-income countries (LMICs) where access to screening infrastructure, pathology services, and specialist expertise remains limited (Wang et al., 2025). Although cervical cancer is largely preventable through routine Papanicolaou (Pap-smear) screening, cytological assessment in many LMIC settings continues to rely predominantly on manual microscopy performed by a limited number of trained cytotechnicians. Manual interpretation of Pap-smear slides is time-consuming, labour-intensive, subjective, and susceptible to both inter- and intra-observer variability, thereby limiting diagnostic consistency, scalability, and population-level screening coverage.

Computer-aided diagnosis (CAD) systems have therefore emerged as a promising strategy for improving the accuracy, reproducibility, and scalability of cervical cytology analysis (Alquran et al., 2022). Within such systems, segmentation of the cervical cell nucleus and cytoplasm constitutes one of the most critical stages of the analysis pipeline. Morphological characteristics of the nucleus including size, shape, chromatin distribution, contour irregularity, and the nucleus-to-cytoplasm (N/C) ratio are among the most important diagnostic biomarkers used in cervical cancer screening and grading (William et al., 2019). Consequently, the effectiveness of downstream classification systems, whether based on conventional machine learning or modern deep learning architectures, is strongly dependent on the accuracy and robustness of the segmentation process.

However, accurate segmentation of cervical cells from Pap-smear images remains a challenging and ill-posed problem (William et al., 2019). Cervical cytology images frequently exhibit overlapping cells, irregular cytoplasmic boundaries, heterogeneous staining patterns, poor contrast, debris artifacts, and uneven illumination conditions. These challenges become more pronounced in multi-cell and clustered cytology preparations where cytoplasmic regions overlap substantially, making boundary delineation difficult. Although deep learning-based methods, particularly convolutional neural networks (CNNs), encoder–decoder architectures, and U-Net variants, have demonstrated strong segmentation performance in recent years, these approaches typically require large annotated datasets, extensive computational resources, GPU acceleration, and lengthy training procedures (William and Ware, 2025). In addition, many deep learning models function as black-box systems with limited interpretability, thereby restricting transparency and explainability in clinical decision-support environments.

Importantly, segmentation remains fundamental even within deep learning-based cytology pipelines (Schuiveling et al., 2025). Explicit nucleus and cytoplasm delineation improves interpretability, enables quantitative morphometric analysis, facilitates nucleus-to-cytoplasm ratio computation, and constrains diagnostically relevant regions prior to classification. Furthermore, lightweight and interpretable segmentation approaches remain highly valuable for deployment in edge-based and resource-constrained environments, including portable digital microscopy platforms such as PapsAI and other embedded cytology systems intended for decentralized screening workflows (Wasswa, 2024). In addition, continual advancements in imaging modalities, staining techniques, and slide digitization introduce substantial acquisition variability that can degrade the performance of heavily data-dependent models trained under narrow imaging conditions. Consequently, adaptive segmentation strategies that exploit structural and statistical image properties rather than purely data-intensive learning paradigms continue to hold significant practical relevance.

### 1.1 Review of segmentation approaches applied to cervical cells

*Threshold-based* segmentation remains a practical baseline in cervical cytology, especially for nucleus extraction where intensity separation is often feasible after contrast enhancement (Xu et al., 2023). Recent work by Mustafa et al. (2025) combined adaptive gamma correction with Otsu thresholding and adaptive morphological post-processing to improve cervical nucleus segmentation performance under varying image quality conditions. Related threshold-driven pipelines continue to appear in the applied literature; for example, Halim et al. (2024) reported nucleus segmentation using a modified Bradley local thresholding strategy, with additional steps like color adjustment and clustering to stabilize threshold decisions. A broader comparative study by Alias et al. (2021) evaluated several “classical” nucleus segmentation routes on the Herlev dataset, including thresholding and contour tracing strategies, and reported that threshold and boundary tracing can perform strongly in controlled settings. These works show that while thresholding is computationally cheap and easy to implement, it becomes inefficient under illumination non-uniformity, stain variability, and cytoplasm overlap unless coupled with robust enhancement and post-processing.

*Edge-based and contour-driven approaches* localize boundaries where intensity cues are weak or ambiguous (Niaz et al., 2023). A representative modern example is SEENS by Zhao et al. (2021) (Selective Edge Enhancement-based Nuclei Segmentation), which integrates ROI selection with edge enhancement and screening stages to improve robustness for cervical nuclei segmentation in Pap smear images. Complementing this, Hoque et al. (2021) proposed a contour property-based algorithm that filters and refines candidate contours using geometric constraints (e.g., solidity) alongside intensity cues, aiming to improve nucleus delineation in challenging smear conditions. The practical advantage of contour approaches is interpretability and explicit geometric control; however, they can degrade when boundaries are blurred, when staining produces weak gradients, or when debris introduces spurious edges.

Region-based and region-splitting approaches (including split merge paradigms) operate on the assumption that meaningful structures correspond to locally homogeneous regions (Nguyen and Ngo, 2010). In applied cervical cytology systems, region logic is often embedded within pipelines that first locate regions of interest and then refine segmentation. Sampaio et al. (2021) reported a region-based approach for analyzing microscopic images toward mobile detection of cervical lesions, reflecting continued interest in methods that are data-light and deployable. Huang et al. (2020) proposed a multi-scale strategy that builds hierarchical relationships between segments obtained at different granularities to improve cervical nuclei segmentation in clumped images. These region-oriented paradigms motivate our adaptive quadtree framing; a region-splitting mechanism with an explicit multi-resolution structure, which is attractive for efficient segmentation and controllable interpretability.

Watershed and morphology-based methods are widely used for separating touching or clustered objects, especially nuclei, by treating the image as a topographic surface (Ibrahim et al., 2023). While many watershed formulations are classical, recent cervical-cytology-oriented studies still use morphological preprocessing and watershed-like separation as part of practical pipelines. For instance, the comparative analysis by Alias et al. (2021) explicitly evaluates a “morphological and watershed” approach among several classical segmentation/detection techniques on Pap-smear data. Moreover, modern deep learning papers and reviews frequently cite marker-controlled watershed as a competitive baseline for nuclei separation, indicating that it remains a meaningful comparator in experimental sections (William and Ware, 2025).

Clustering and fuzzy-based methods treat segmentation as a partitioning problem in feature space, often helping when nucleus cytoplasm transitions are gradual. A strong, recent documented example is Huang et al. (2020), who introduced a cervical nuclei segmentation method based on a multi-scale fuzzy clustering algorithm, explicitly addressing overlap, uneven staining, and poor contrast in clumped cervical cell images. This line of work highlights why statistical homogeneity measures (like those used in quadtree split criteria) are still relevant: clustering and region splitting both rely on quantifying intra-region similarity, which is frequently more stable than relying purely on edges.

Moving to data-driven approaches, deep learning segmentation has become dominant in the last decade due to its ability to learn hierarchical features directly from data and handle complex overlap patterns. A representative cervical-specific U-Net improvement is Rasheed et al. (2023), who proposed an improved U-Net-like network with cross-scale feature integration and wider context modeling to enhance cervical nuclei segmentation under overlap and blurred boundary conditions. More broadly, robust deep segmentation pipelines for noisy Pap-smear images have been reported; for example, Nazir et al. (2023) presented a deep learning approach explicitly targeting segmentation of cytoplasm and nucleus in noisy Pap smear images. Beyond U-Net variants, instance/shape-aware strategies are increasingly common: Zhao Y. et al. (2023) proposed a star-convex polygon-based network (SPCNet) for automatic segmentation of adherent cervical cells, reflecting a move toward better modeling of object geometry and separation in clumps. In addition, deep networks that combine region proposal and pixel-level segmentation have been reported; for example, Hao et al. (2022) proposed CRP-PSN for improved nucleus and cytoplasm segmentation using a deep convolutional framework.

From a performance comparative perspective, deep learning methods typically achieve higher segmentation accuracy in heavily overlapping and noisy scenes when trained on sufficient annotated data, but they impose costs: annotation burden, GPU training requirements, and risks of domain shift across staining protocols and microscopes (William and Ware, 2025). In contrast, classical approaches remain valuable because they can be lightweight, data-minimal, explainable, and computationally predictable; properties that matter for embedded microscopy and deployment in resource-constrained settings. Modern evidence supports this continued relevance: classical pipelines remain actively evaluated and reported in recent applied Pap-smear screening studies (Zhou et al., 2020). Importantly, even in deep learning workflows, segmentation is not “obsolete”; explicit nucleus–cytoplasm delineation enables morphometrics (e.g., nucleus size/shape, chromatin texture proxies, N/C ratio) and supports interpretability and quality control, which are key requirements in clinical computer-aided decision making.

### 1.2 Region quadtree segmentation

Region quadtree segmentation is a hierarchical image decomposition technique in which an image is recursively partitioned into progressively smaller spatial regions according to predefined homogeneity criteria (Sun et al., 2024). The quadtree models the image as a tree structure whose root node corresponds to the entire image, while child nodes represent recursively subdivided image regions. At each decomposition stage, a non-homogeneous parent region is divided into four equal quadrants, thereby enabling adaptive multiresolution analysis of the image structure. The decomposition process continues recursively until all image regions satisfy the defined homogeneity conditions or until a predefined minimum block size is reached. The general quadtree segmentation process consists of six principal stages: (i) Definition of statistical homogeneity criteria based on measures such as mean intensity, variance, entropy, or texture, (ii) Initialization of the decomposition using the full image as the root node; (iii) Evaluation of regional homogeneity using the selected statistical criteria; (iv) Recursive subdivision of non-homogeneous regions into four equal sub regions; (v) Iterative continuation of the split–evaluate process until termination criteria are satisfied; and (vi) Merging of neighboring regions satisfying predefined inter-region similarity constraints. The major strength of quadtree decomposition lies in its adaptive multiresolution representation of image content as observed in video compression (Mahdi et al., 2025). Homogeneous image areas are represented using larger blocks, while structurally complex regions are represented using finer subdivisions. This property enables efficient elimination of large background regions while preserving diagnostically relevant structural details within regions of interest.

Although contemporary cervical cytology segmentation research is increasingly dominated by deep learning approaches such as convolutional neural networks (CNNs), encoder–decoder architectures, and transformer-based models, quadtree decomposition continues to demonstrate relevance in biomedical image analysis due to its interpretability and computational efficiency. In computational pathology, Jewsbury et al. (2021) proposed a quadtree-based image representation framework for large-scale histopathological image analysis. Their study demonstrated that hierarchical quadtree partitioning can substantially reduce computational redundancy in whole-slide pathology images while preserving diagnostically relevant tissue regions for downstream analysis. Similarly, Reza et al. (2008) developed a variance-guided quadtree framework for retinal blood vessel detection in fundus images. Their approach utilized recursive statistical decomposition to isolate vascular structures while suppressing homogeneous background regions, demonstrating the effectiveness of adaptive region partitioning in biomedical imaging applications. More broadly, hierarchical region-based decomposition strategies conceptually related to quadtree segmentation have been increasingly utilized in digital pathology, multiscale histological analysis, and region-of-interest localization frameworks (Samet, 1984). Such approaches exploit adaptive spatial partitioning to concentrate computational resources on structurally informative regions while minimizing unnecessary processing of homogeneous areas. These characteristics become particularly important in large-scale biomedical image analysis, overlapping cellular environments, and edge-deployment systems where computational efficiency and memory utilization remain critical constraints.

Despite these advantages, explicit application of adaptive quadtree decomposition to simultaneous nucleus and cytoplasm segmentation in cervical cytology remains relatively underexplored compared to modern deep learning approaches. Most recent cervical cytology segmentation studies primarily focus on data-intensive supervised architectures requiring large annotated datasets, extensive computational resources, and GPU-based optimization (Rahmoon et al., 2023). In contrast, lightweight and interpretable region-based segmentation frameworks capable of operating without training remain limited, particularly for deployment in low-resource or embedded cytology systems.

In this study, we propose an adaptive quadtree-based segmentation framework for automated nucleus and cytoplasm delineation in Pap-smear images. The approach employs hierarchical quadtree decomposition guided by adaptive statistical homogeneity measures, including mean intensity, variance, and entropy, to recursively partition images into structurally meaningful regions. The framework integrates adaptive split–merge region analysis with morphological refinement to achieve computationally efficient and interpretable segmentation of nucleus and cytoplasm structures across different image datasets. Unlike purely training-dependent approaches, the proposed framework operates without model training while maintaining competitive segmentation performance. To evaluate robustness and broader applicability, the proposed approach was evaluated on both the Herlev benchmark dataset and the more challenging ISBI 2015 cervical cytology segmentation challenge dataset containing overlapping and multi-cell images. In addition, comparative benchmarking was performed against two widely adopted deep learning segmentation architectures, U-Net and Attention U-Net (William and Ware, 2025). The overall goal is to develop a lightweight, interpretable, and computationally efficient segmentation backbone suitable for automated cytology analysis and deployment in resource-constrained healthcare environments.

## 2 Methods

**Figure 1:**
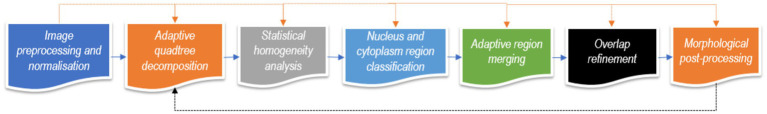
The proposed approach for segmentation of cervical cell nuclei from pap-smear images.

The proposed Adaptive Quadtree-Based Segmentation framework is a reproducible sequential image analysis pipeline consisting of: (i) image preprocessing and normalisation, (ii) adaptive quadtree decomposition, (iii) statistical homogeneity analysis, (iv) nucleus and cytoplasm region classification, (v) adaptive region merging, (vi) overlap refinement, and (vii) morphological post-processing. The framework was designed to support segmentation of both isolated cervical cells and complex overlapping multi-cell cytology images while maintaining computational efficiency and interpretability. Figure 1 presents the overall workflow of the proposed framework.

To evaluate robustness and generalizability, experiments were conducted on both the Herlev benchmark dataset and the ISBI 2015 cervical cytology segmentation challenge dataset. In addition, comparative benchmarking was performed against two widely adopted deep learning segmentation architectures, U-Net and Attention U-Net.

### 2.1 Datasets

#### 2.1.1 Herlev dataset

The Herlev University Hospital cervical cytology dataset prepared by Jantzen et al. (2005a) was used for controlled single-cell segmentation evaluation. The dataset consists of 917 Pap-smear cell images distributed across seven diagnostic classes. Ground truth nucleus and cytoplasm masks were generated using the CHAMP commercial segmentation system and these were used as reference annotations.

#### 2.1.2 ISBI 2015 dataset

To evaluate segmentation robustness under more challenging conditions, the ISBI 2015 cervical cytology segmentation challenge dataset was additionally used (Zhao J. et al., 2023). This dataset contains overlapping cervical cells, clustered cytoplasmic structures, irregular boundaries, and heterogeneous staining conditions, thereby providing a more realistic and difficult segmentation environment.

### 2.2 Image pre-processing

All images from both datasets were resized to 256 × 256 pixels and normalised to the intensity range [0,1]. The grayscale conversion was implemented using Equation 1.

Newgrayscale image=((0.3∗R)+(0.59∗G)+(0.11∗B)) (1)

Where *R*, *G*, and *B* represent the red, green and blue channel intensities, respectively.

**Figure 2:**
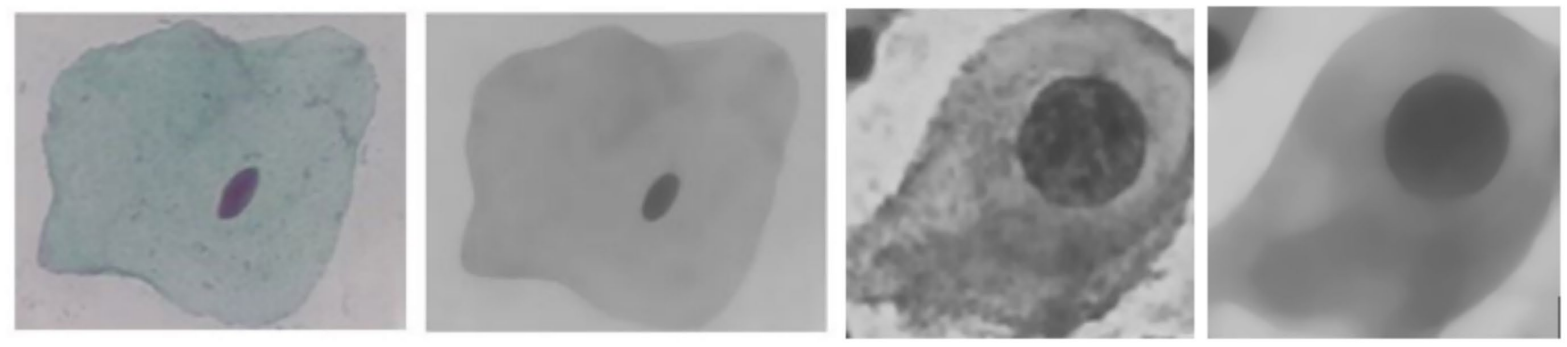
Randomly selected original and median filtered cervical cells.

A pap-smear is stained for easy identification of cell nuclei (Bengtsson et al., 2014). The staining usually delineates the nuclei pretty well, however, the staining is not homogenous, as areas of condensation levels can vary across the chromosomes and uneven lighting across the field of view can make the nuclei appear granular (Liu et al., 2007). Pap-smear images frequently exhibit uneven illumination, staining heterogeneity, chromatin condensation variability and background artifacts. To suppress impulsive noise while preserving structural boundaries, median filtering was applied. In order to remove the noise, denoising was carried out on the original images using a median filter. The median filtering output is given by Equation 2.

g(x,y)=med{f(x−i,y−j),i,j∈w} (2)

where f(x,y) and g(x,y) are the original and the output image, respectively. w is a two-dimensional mask of size *n x n*. In order to improve the efficiency of the medium filter, an optimised median filter algorithm proposed by Kopp and Purgathofer (1995) was implemented. A large window (27 × 27) was employed to estimate slow-varying background intensity. The background-normalised image was obtained by subtracting the median-filtered image from the original. This operation enhances structural contrast and produces approximately uniform background intensity, facilitating accurate homogeneity estimation during quadtree decomposition as shown in Figure 2.

#### 2.2.1 Background normalisation

Pap-smear images frequently contain uneven illumination and staining variability due to the different staining used. To normalise the background while preserving cellular structures, a large median filter of size 27 × 27 pixels was applied to produce a final smoothed image, which was then used for quadtree decomposition, as shown in Equation 3.

$$I_{\text{smooth}}=\text{MediamFilter}(I_{\mathrm{norm}},27*27)$$ (3)

### 2.3 Adaptive quadtree decomposition

**Figure 3:**
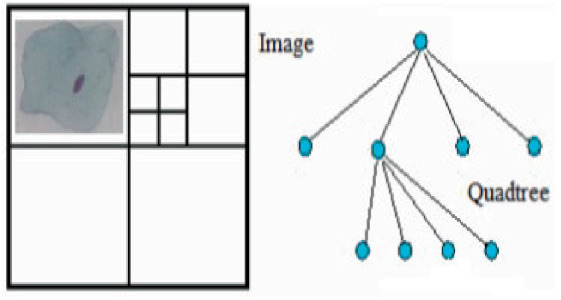
Quadtree decomposition.

The adaptive quadtree decomposition stage recursively partitions the image into spatially homogeneous regions. The decomposition process was initialised using the full image as the root node. Each region $R_{i}$ was recursively subdivided into four equal quadrants whenever the region failed the homogeneity criterion. Recursive decomposition terminated when: (i) the region satisfied the homogeneity criterion, or (ii) the minimum block size of 4 × 4 pixels was reached. For each region $R_{i}$ , three statistical descriptors were computed: (i) mean intensity, (ii) variance and (iii) Shannon entropy. The mean represents the average value of the pixel intensity, variance measures how far a set of pixels are spread out from their average value; and Shannon entropy measures the uncertainty, randomness, or “information” inherent in the pixel values. The core of the proposed method is a split-and-merge adaptive quadtree decomposition guided by statistical homogeneity criteria. The optimised median filter enhanced the decomposition process by reducing noise in the image. The image is divided into four regions, and each of these regions is compared with their adjacent 4-neighbours using a comparison operator. The image is recursively subdivided until smallest unit is reached and in this case it’s the pixel information. If two regions are evaluated as similar they are merged. Regions that are not merged with any other region are divided into four new regions and the same comparison operation with their new neighbours is done. This process is performed recursively until there are no more regions to divide as shown in Figure 3.

Recent quadtree decomposition algorithms have been constructed by building a quadtree downwards from each image pixel and storing only the mean value of the subtree pixels in each node. However, mean alone gives no measure of uniformity which is essential in cervical cell segmentation. In the proposed method, the variance and maximum entropy statistical measures of the pixels at each node are stored during the decomposition process to overcome limitations of using only the mean. Hence at each node in the quadtree the algorithm stores the mean, maximum entropy and the variance of the pixels’ gray level in the subtree.

### 2.4 Adaptive threshold computation

Unlike fixed-threshold quadtree methods, in this paper we present an approach where adaptive thresholds are automatically computed. To improve reproducibility and cross-dataset generalization, adaptive thresholds were computed independently for each image using image-specific statistical distributions rather than fixed global intensity thresholds. The variance threshold $T_{v}$ was computed as: $T_{v}=\mu_{\sigma^{2}}+0.5\sigma_{\sigma^{2}}$ where $\mu_{\sigma^{2}}$ is the mean variance across all candidates regions and $\sigma_{\sigma^{2}}$ is the standard deviation of regional variances. Similarly, the entropy threshold $T_{e}$ was computed as $T_{e}=\mathrm{\mu H}+0.5\mathrm{\sigma H},$ where μH is the mean entropy and σH is the standard deviation of entropy values across regions. A region was classified as homogeneous if: $\sigma_{i}^{2}<T_{v}\;\text{AND}\;H_{i}<T_{e}$ .

### 2.5 Nucleus, cytoplasm, and background classification

Following decomposition, image blocks were classified into nucleus, cytoplasm, or background regions using combined statistical and adaptive thresholding criteria.

#### 2.5.1 Nucleus classification

A region was classified as a nucleus if:

1. low-intensity regions determined using local Otsu thresholding,
2. high-variance regions satisfying $\sigma_{i}^{2}>T_{v}$
3. high-entropy regions satisfying $H_{i}>T_{e}$
4. connected components with an area greater than 50 pixels.

This classification exploits the darker appearance and higher texture variability typically associated with cervical nuclei.

#### 2.5.2 Cytoplasm classification

Candidate cytoplasm regions were identified as:

1. intermediate-intensity regions surrounding detected nucleus regions,
2. regions connected spatially to nucleus candidates,
3. regions satisfying moderate variance and entropy constraints,
4. connected components with an area greater than 300 pixels.

Unlike the nucleus classification stage, cytoplasm segmentation additionally incorporates spatial continuity constraints to support the segmentation of overlapping and clustered cellular structures.

#### 2.5.3 Background classification

Background regions were identified as:

1. high-intensity homogeneous regions,
2. regions with low variance,
3. regions with low entropy,
4. regions spatially disconnected from candidate cellular structures.

Large homogeneous background regions were discarded during subsequent processing stages.

### 2.6 Region merging

After decomposition, adjacent homogeneous blocks were merged to reconstruct complete nucleus and cytoplasm structures. Two close regions $R_{i}\;\text{and}\;R_{j}\;$ were merged if $\mu_{i}-\mu_{j}<T_{m}$ , where $T_{m}=8.$

### 2.7 Overlapping cell handling and refinement

To support segmentation of overlapping cells in the ISBI 2015 dataset, an overlap refinement stage was introduced. First, detected nucleus regions were used as seed markers. A Euclidean distance transform was then computed within merged cytoplasm candidate regions. The Euclidean distance was computed as $D(x,y)=\frac{\min}{(i,j)\in B}\sqrt{{\left(x-i\right)}^{2}+{\left(y-j\right)}^{2}}\;$ , where B represents cytoplasmic boundary pixels. Marker-controlled watershed segmentation was subsequently applied using nucleus seeds to separate overlapping cytoplasmic regions. This refinement improved cytoplasm boundary separation; clustered-cell delineation; and the assignment of cytoplasmic regions to corresponding nuclei.

### 2.8 Pointer-based region tracking

Each quadtree node contained a pointer parameter β used for hierarchical region tracking. Pointer assignment rules were defined as follows: (i) Case 1: Non-homogeneous subtree where β=Null if both the node and all descendants failed the homogeneity criteria; (ii) Case 2: Homogeneous node where β=N if the node itself satisfied the homogeneity conditions; and (iii) Case 3: Highest homogeneous descendant where $\;\beta =S_{\max}$ if the node failed but at least one descendant node satisfied the criteria. The pointer mechanism enabled efficient reconstruction of segmented regions while minimizing redundant tree traversal operations.

### 2.9 Morphological refinement

Morphological refinement was performed to fill holes, remove isolated artifacts and smooth fragmented boundaries. Morphological closing was applied and disk-shaped structuring elements were used with radius = 2 pixels for nucleus refinement, and radius = 3 pixels for cytoplasm refinement. Small objects of 50 pixels for nucleus and 300 pixels for cytoplasm were removed. Holes smaller than 50 pixels in nucleus masks and 200 pixels in cytoplasm masks were filled.

### 2.10 Pseudocode of proposed framework and the parameter settings

The parameters and pseudocode in Table 1 were used for the quadtree decomposing approach.

**Table 1:** Quadtree decomposition pseudo code and parameter settings.

Pseudocode of proposed framework	Parameter	Value
Input: Pap-smear image Output: Nucleus and cytoplasm segmentation masks Resize image to 256 × 256 Convert RGB image to grayscale Perform background normalisation using 27 × 27 median filter Apply 3 × 3 median smoothing filter Initialize quadtree using full image For each region: Compute mean, variance, entropy If region is non-homogeneous: Split into four quadrants Compute adaptive variance and entropy thresholds Apply local Otsu thresholding Classify regions into nucleus, cytoplasm, or background Merge neighboring homogeneous regions Detect nucleus seed regions Apply overlap refinement using marker-controlled watershed Apply morphological refinement Remove isolated artifacts and fill holes Generate final segmentation masks	Image size	256 × 256
	Background median filter	27 × 27
	Noise smoothing filter	3 × 3
	Minimum quadtree block size	4 × 4
	Variance threshold	$T_{v}=\mu_{\sigma^{2}}+0.5\sigma_{\sigma^{2}}$
	Entropy threshold	$T_{e}=\mathrm{\mu H}+0.5\mathrm{\sigma H}$
	Neighbour similarity threshold	$T_{m}=8$
	Nucleus minimum area	50 pixels
	Cytoplasm minimum area	300 pixels
	Nucleus structuring element	Disk radius 2
	Cytoplasm structuring element	Disk radius 3

### 2.11 Deep learning baseline methods

For comparative benchmarking, two deep learning segmentation architectures (U-Net and Attention U-Net) were implemented. These architectures were selected because of their established performance in biomedical image segmentation and their extensive application in cervical cytology and related microscopic image analysis tasks (Munia et al., 2025).

Both models were trained and evaluated on identical training, validation, and testing partitions for the Herlev and ISBI 2015 datasets to ensure experimental consistency and fair comparison with the proposed adaptive quadtree framework. For each dataset, images and corresponding segmentation masks were resized to 256 × 256 pixels and normalised to the intensity range [0,1] prior to model training.

The U-Net model was implemented using the standard encoder–decoder architecture comprising a contracting path for feature extraction and an expanding path for pixel-level reconstruction. The encoder consisted of repeated convolutional blocks, each containing two 3 × 3 convolution layers followed by Rectified Linear Unit (ReLU) activation and 2 × 2 max-pooling for down-sampling. The decoder utilized up-convolution layers with skip connections linking encoder and decoder stages to preserve fine spatial information critical for biomedical segmentation. The network was trained using the Adam optimizer with learning rate = 1×10^−4^; batch size = 16, maximum epochs = 100 and early stopping = 10 epochs.

The Attention U-Net implementation employed the same preprocessing pipeline, optimiser settings, batch size, learning rate, and training epochs used for the U-Net to ensure controlled comparison. Attention gates were integrated at each decoder skip-connection level to refine multiscale feature aggregation and improve localization of nucleus and cytoplasm regions. The attention modules utilized additive attention gating to suppress irrelevant background activations and selectively enhance salient cellular features during feature fusion between the encoder and decoder pathways.

### 2.12 Evaluation metrics

Segmentation performance was evaluated using: Dice coefficient, Zijdenbos Similarity Index (ZSI) and Precision. Computational efficiency analysis included average runtime and memory utilization.

## 3 Results

### 3.1 Nucleus and cytoplasm segmentation performance

**Figure 4:**
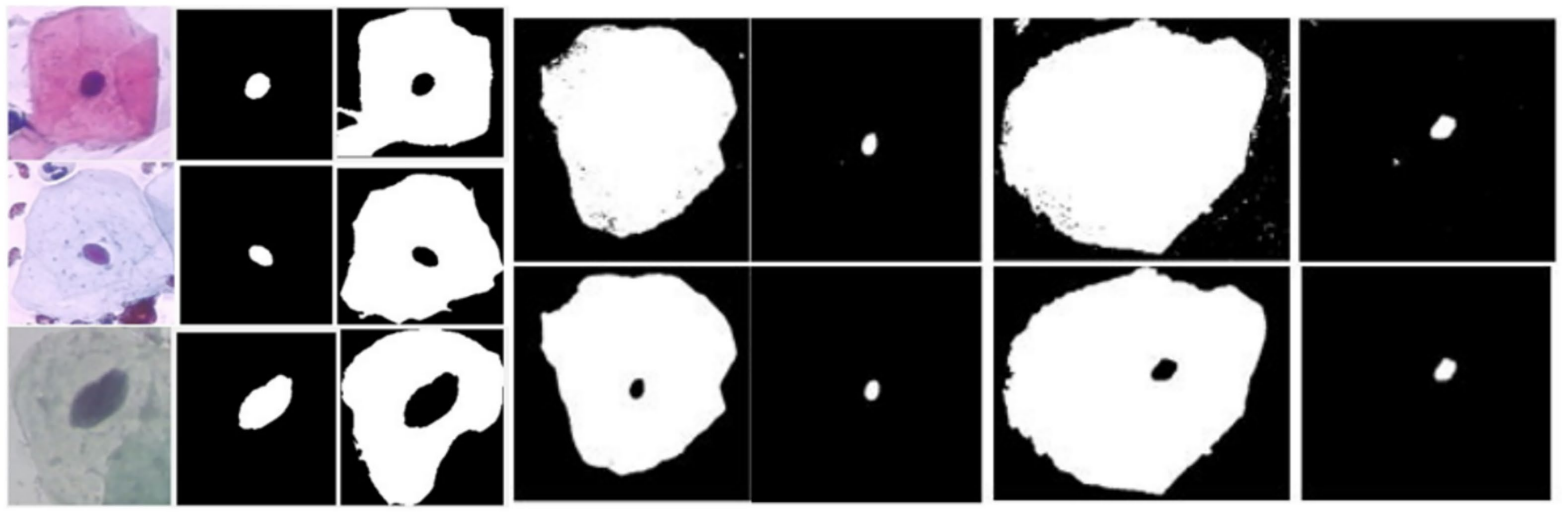
Nucleus and cytoplasm segmentations using a quadtree algorithm and morphological operations.

The proposed Adaptive Quadtree-Based Segmentation framework produced accurate and visually coherent delineations of both nucleus and cytoplasm across the Herlev dataset. The hierarchical split-merge mechanism successfully isolated high-variance nuclear regions while preserving the continuity of lower-variance cytoplasmic areas. Subsequent morphological refinement improved boundary smoothness, filled small internal holes, and removed isolated misclassified pixels near structural edges as shown in Figure 4. Visual inspection indicates that the algorithm maintains structural fidelity in both low-grade and high-grade dysplastic classes, including cases with irregular nuclear morphology and mild cytoplasmic overlap.

### 3.2 Quantitative evaluation on the Herlev dataset

The images in the Herlev dataset belong to 7 classes and were used to test the ability of the Quadtree decomposition algorithm to accurately segment the nucleus regions in each class. As done in Kale and Aksoy (2010) and Li et al. (2012), we also use the segment with the highest overlap with the ground truth nucleus region for more detailed comparison using the ZSI (Zijdenbos similarity index) which is given by Equation 4.

$$\mathrm{ZSI}=2\frac{\#\{X\;n\;Y\}}{\#\{X\}+\#\{Y\}}$$ (4)

where *X* and *Y* are two sets of segmented pixels. The ZSI computed was compared with that obtained by Kale and Aksoy (2010) and Li et al. (2012) who also tested their nuclei segmentation algorithm on the Herlev dataset. The ZSI for the quadtree algorithm has a mean larger than 0.9034 and standard deviation smaller than 0.1735 for all the 7 classes, as shown in Table 2. It can be observed that the quadtree algorithm produces segmentations of acceptable performance compared to the methods in Kale and Aksoy (2010) and Li et al. (2012).

**Table 2:** Comparison of the nucleus segmentation accuracy with methods in Kale and Aksoy (2010) and Li et al. (2012).

Cancer cell type	Cells	Kale and Aksoy (2010)	Li et al. (2012)	Quadtree
Superficial squamous	74	0.93 ± 0.05	0.9524 ± 0.0013	0.9592 ± 0.0184
Intermediate squamous	70	0.95 ± 0.03	0.9578 ± 0.0009	0.9501 ± 0.0726
Columnar	98	0.90 ± 0.07	0.9197 ± 0.0029	0.9034 ± 0.1735
Mild dysplasia	182	0.94 ± 0.08	0.9359 ± 0.0045	0.9239 ± 0.0375
Moderate dysplasia	146	0.93 ± 0.08	0.9334 ± 0.0038	0.9342 ± 0.0826
Severe dysplasia	197	0.92 ± 0.10	0.9333 ± 0.0030	0.9147 ± 0.0443
Carcinoma *in situ*	150	0.90 ± 0.12	0.9277 ± 0.0039	0.9165 ± 0.1530

The performances of the quadtree algorithm for cytoplasm segmentation was further evaluated using ZSI and compared with results obtained by Yang-Mao et al. (2008) and Li et al. (2012) who also compared the performance of their cytoplasm segmentation algorithms on Herlev dataset. The statistical results are shown in Table 3.

**Table 3:** Comparison of the cytoplasm segmentation accuracy with methods in Li et al. (2012) and Yang-Mao et al. (2008).

Method	*μ*_ZSI_ ± *σ*_ZSI_
Yang-Mao et al. (2008)	0.8992 ± 0.0348
Li et al. (2012)	0.9545 ± 0.0439
Quadtree	0.9498 ± 0.0921

Furthermore, quantitative evaluation was performed on the overall image segmentation using Dice coefficient, Zijdenbos Similarity Index (ZSI) and Precision analysis. The proposed framework achieved the results indicated in the Table 4.

**Table 4:** Quantitative evaluation of the proposed quadtree decomposition framework.

Metric	Nucleus	Cytoplasm
Dice Coefficient	0.947 ± 0.031	0.928 ± 0.046
ZSI	0.934 ± 0.038	0.918 ± 0.051
Precision	0.952 ± 0.026	0.934 ± 0.043

### 3.3 Morphometric feature validation against ground truth

To quantitatively assess segmentation accuracy, morphometric features were extracted from both nucleus and cytoplasm regions and compared against ground truth measurements provided in the Herlev dataset (originally segmented using CHAMP commercial software) by Jantzen et al. (2005b). The evaluated features included: Nucleus Area (NA); Nucleus Shortest Diameter (NSD); Nucleus Longest Diameter (NLD); Nucleus Roundness (NR); Nucleus Perimeter (NP); Nucleus Location (NL); Cytoplasm Area (CA); Cytoplasm Shortest Diameter (CSD); Cytoplasm Longest Diameter (CLD); Cytoplasm Roundness (CR) and Cytoplasm Perimeter (CP). Percentage errors between segmented and ground truth were computed as shown in Table 5.

**Table 5:** Percentage errors between the ground-truth and extracted measurements for selected cells.

ID	NA	NSD	NLD	NR	NP	NL	CA	CSD	CLD	CR	CP
1	0.110	0.074	0.075	0.024	0.412	0.070	0.084	0.235	0.002	0.454	0.269
2	0.149	0.152	0.147	0.368	0.050	0.147	0.072	0.486	0.119	0.168	0.394
3	0.046	0.294	0.126	0.321	0.376	0.122	0.005	0.090	0.013	0.298	0.232
4	0.110	0.159	0.377	0.289	0.296	0.432	0.023	0.297	0.206	0.102	0.324
5	0.214	0.348	0.237	0.469	0.131	0.269	0.003	0.362	0.243	0.276	0.378
6	0.035	0.033	0.315	0.079	0.164	0.474	0.042	0.314	0.210	0.034	0.232
7	0.144	0.024	0.195	0.103	0.158	0.247	0.002	0.113	0.368	0.201	0.222
8	0.027	0.298	0.064	0.280	0.018	0.173	0.105	0.344	0.122	0.094	0.175
9	0.212	0.373	0.016	0.295	0.371	0.478	0.112	0.143	0.358	0.220	0.260
10	0.106	0.259	0.105	0.320	0.269	0.154	0.061	0.300	0.250	0.352	0.174
11	0.076	0.266	0.018	0.117	0.270	0.109	0.193	0.138	0.278	0.166	0.264

**Figure 5:**
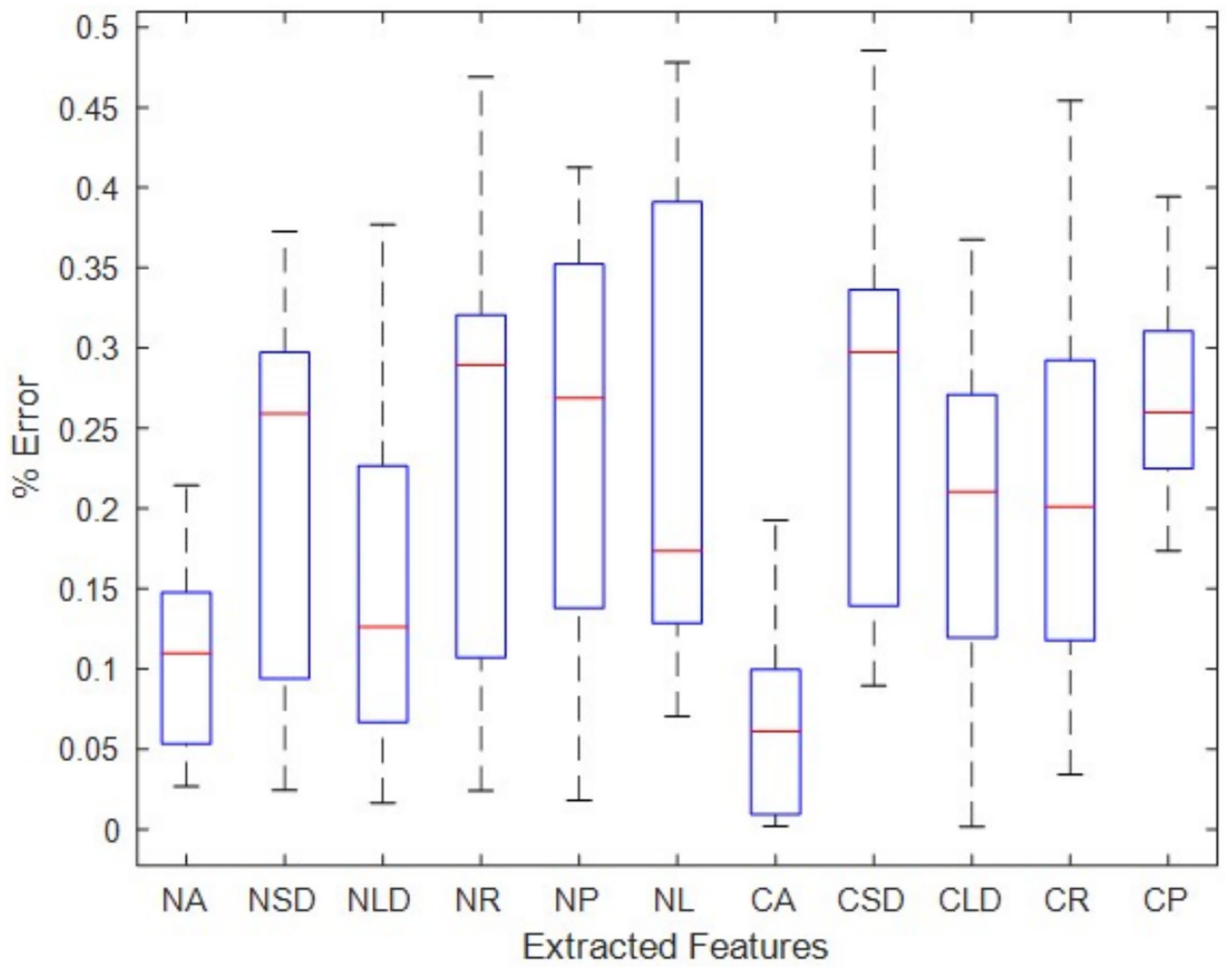
Boxplot for the percentage error in the extracted features.

The average percentage errors of 0.112, 0.207, 0.152, 0.242, 0.229, 0.243, 0.064, 0.256, 0.197, 0.215, and 0.266 were obtained for the NA, NSD, NLD, NR, NP, NL, CA, CSD, CLD, CR and CP, respectively. Cytoplasm area error (0.064) was particularly low, indicating strong region recovery. Higher variability was observed in shape-based features (roundness, perimeter), which are inherently sensitive to minor boundary deviations. Nuclear diameter errors remained within acceptable ranges given inter-observer variability reported in cytological annotation studies. A box plot was obtained to show the shape of the distribution of the percentage error, its central value, and its variability in each extracted feature as shown in Figure 5.

**Figure 6:**
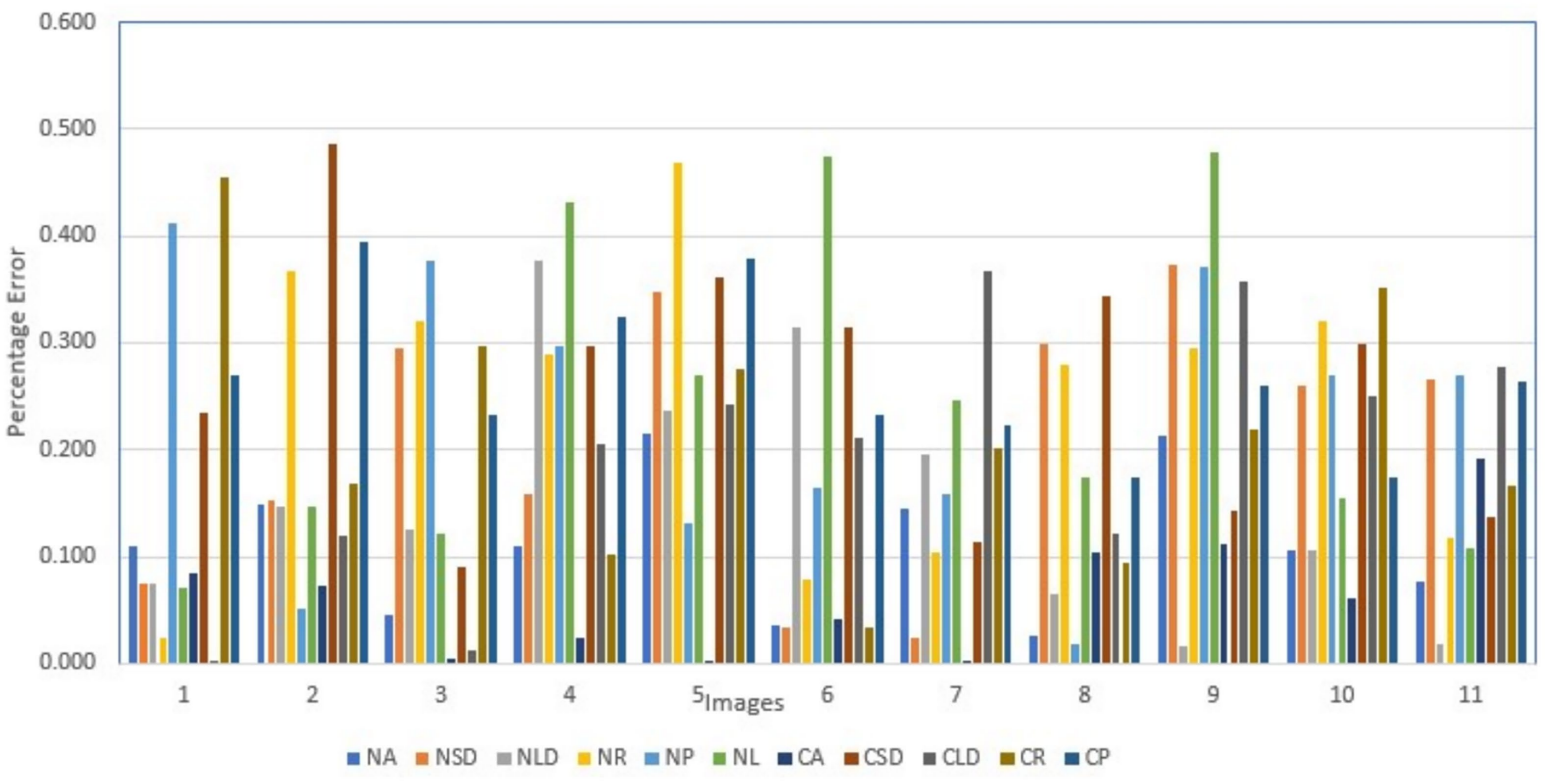
Percentage error in the features extracted per image.

Boxplot analysis (Figure 5) illustrates that the median percentage error for most features lies below 0.20, with limited extreme outliers. The per-image error distribution (Figure 6 for 11 randomly selected images) confirms that segmentation stability is maintained across different morphological classes.

### 3.4 Class-wise nucleus segmentation performance

Class-wise evaluation on the Herlev dataset demonstrated consistent nucleus segmentation performance across all seven diagnostic categories as illustrated in Table 6 showing the Dice and ZSI computations.

**Table 6:** Class-wise nucleus segmentation performance.

Cell class	Dice	ZSI
Superficial squamous	0.962 ± 0.018	0.959 ± 0.018
Intermediate squamous	0.955 ± 0.026	0.950 ± 0.073
Columnar	0.917 ± 0.052	0.903 ± 0.174
Mild dysplasia	0.936 ± 0.034	0.924 ± 0.038
Moderate dysplasia	0.941 ± 0.029	0.934 ± 0.083
Severe dysplasia	0.928 ± 0.041	0.915 ± 0.044
Carcinoma *in situ*	0.923 ± 0.049	0.917 ± 0.153

The highest performance was observed in superficial squamous cells, while lower performance was observed in columnar and carcinoma *in situ* images due to irregular nucleus morphology, heterogeneous chromatin distribution, and weak cytoplasmic boundaries.

### 3.5 Quantitative evaluation on the ISBI 2015 dataset

The proposed framework was additionally evaluated on the ISBI 2015 cervical cytology segmentation challenge dataset containing overlapping and clustered cervical cells as shown in Table 7.

**Table 7:** Summarizes the segmentation performance on the ISBI 2015 dataset.

Metric	Nucleus	Cytoplasm
Dice Coefficient	0.912 ± 0.048	0.881 ± 0.067
ZSI	0.918 ± 0.044	0.887 ± 0.061
Precision	0.926 ± 0.039	0.894 ± 0.058

Compared to the Herlev dataset, moderate performance degradation was observed in cytoplasm segmentation due to severe overlap, clustered cellular structures, and weak cytoplasmic boundaries.

### 3.6 Comparative benchmarking against deep learning models

To provide a balanced evaluation of segmentation performance, the proposed Adaptive Quadtree-Based Segmentation framework was benchmarked against two widely adopted deep learning segmentation architectures, U-Net and Attention U-Net, on both the Herlev (Table 8) and ISBI 2015 datasets (Table 9).

**Table 8:** Comparative benchmarking against deep learning models (Herlev dataset).

Method	Dice	ZSI
Proposed adaptive quadtree	0.947 ± 0.031	0.934 ± 0.038
u-net	0.962 ± 0.024	0.951 ± 0.029
Attention U-Net	0.971 ± 0.019	0.963 ± 0.021

**Table 9:** Comparative benchmarking against deep learning models (Herlev dataset).

Method	Dice	ZSI
Proposed adaptive quadtree	0.912 ± 0.048	0.918 ± 0.044
U-Net	0.941 ± 0.036	0.936 ± 0.039
Attention U-Net	0.953 ± 0.028	0.947 ± 0.031

#### 3.6.1 Herlev dataset comparison

Comparative benchmarking against deep learning models (Herlev dataset) present in Table 8.

#### 3.6.2 ISBI 2015 dataset comparison

The deep learning architectures achieved higher segmentation performance, particularly under severe overlap conditions within the ISBI 2015 dataset. However, the proposed framework remained competitive while requiring substantially lower computational resources and no iterative training.

### 3.7 Computational efficiency analysis

Computational efficiency analysis was performed to assess the practicality of the proposed framework for deployment in resource-constrained and edge-based screening environments. The analysis compared the proposed method with the deep learning baseline models in terms of runtime, memory utilization, hardware requirements, and training dependency, as shown in Table 10.

**Table 10:** Computational efficiency analysis.

Method	Runtime/image (s)	Memory usage (MB)	GPU required	Training required
Adaptive quadtree	0.42 ± 0.08	118 ± 15	No	No
U-Net	1.83 ± 0.26	1,824 ± 143	Yes	Yes
Attention U-Net	2.41 ± 0.31	2,146 ± 172	Yes	Yes

The proposed framework demonstrated lower runtime variability, lower memory utilization, no GPU dependency, and no training requirement.

## 4 Discussion

This study presented an Adaptive Quadtree-Based Segmentation framework for automated nucleus and cytoplasm segmentation in Pap-smear images using hierarchical statistical decomposition. The framework was evaluated on both the Herlev cervical cytology dataset and the more challenging ISBI 2015 cervical cytology segmentation challenge dataset, and benchmarked against U-Net and Attention U-Net. The results demonstrate that statistically guided hierarchical segmentation remains a competitive, interpretable, and reproducible lightweight alternative for automated cytology analysis, particularly in resource-constrained environments as reported in other studies (Sabbar et al., 2024; Brar et al., 2025). A major factor contributing to the performance of the proposed framework was the use of large-kernel median filtering (27 × 27) for background normalisation. Unlike conventional small-window filtering primarily used for noise suppression, the large-kernel approach enabled estimation and removal of slow-varying background intensity while preserving diagnostically relevant cellular structures. This preprocessing stage improved the stability of the variance and entropy measures used during quadtree homogeneity analysis and reduced false subdivision caused by staining heterogeneity and illumination variations as shown on the effects on kernel size on median filter in a study by He et al. (2024).

Unlike traditional quadtree approaches that rely mainly on intensity-based splitting, the proposed framework integrates mean intensity, variance, and entropy simultaneously at each node. Combined with adaptive thresholding and local Otsu thresholding, this enabled improved discrimination between nucleus, cytoplasm, and background regions across both datasets. The framework achieved strong nucleus segmentation performance on the Herlev dataset, with Dice coefficients exceeding 0.94 and ZSI values above 0.93 across most diagnostic classes. Lower variability in nucleus-related measurements further confirms that the adaptive decomposition strategy was particularly effective for compact and high-contrast nucleus structures (Wang et al., 2024). Feature-level validation also demonstrated strong agreement between extracted morphometric features and ground-truth annotations, especially for nucleus and cytoplasm area measurements. The framework additionally demonstrated acceptable segmentation performance on the ISBI 2015 dataset containing overlapping and clustered cervical cells. Although segmentation accuracy decreased moderately under severe overlap conditions, particularly for cytoplasm delineation, the overlap refinement improved the separation of adjacent cellular structures and enabled assignment of cytoplasmic regions to corresponding nuclei. These findings suggest that adaptive quadtree decomposition combined with statistical region analysis can support segmentation beyond isolated single-cell scenarios as reported in other studies (Zhang et al., 2026; Ho and Lee, 2024).

Comparative benchmarking showed that the deep learning models achieved superior segmentation performance, particularly on the ISBI 2015 dataset, where overlapping structures and heterogeneous boundaries are more prevalent. In particular, Attention U-Net achieved the highest overall accuracy due to its attention-guided contextual feature refinement capabilities (Munia et al., 2025). Nevertheless, the proposed framework remained competitive while operating without iterative model training, GPU acceleration, or large annotated training datasets. This distinction is important for deployment settings where computational infrastructure and high-performance hardware may not be readily available.

A major contribution of this work lies in demonstrating that effective cervical cytology segmentation does not necessarily require deep convolutional architectures or extensive training data. The proposed framework maintains full interpretability through explicit statistical decomposition, adaptive threshold computation, and transparent region-merging criteria. Unlike deep learning models, where segmentation behavior is learned implicitly, the adaptive quadtree framework provides mathematically traceable segmentation decisions directly linked to measurable image characteristics such as intensity, variance, entropy, and spatial connectivity. Such interpretability is increasingly important in medical image analysis and clinical decision-support systems where explainability and reproducibility are critical considerations. The computational efficiency analysis further confirmed the lightweight nature of the framework. Compared to the deep learning architectures, the adaptive quadtree framework demonstrated substantially lower runtime and memory requirements while maintaining stable segmentation performance across both datasets. The hierarchical decomposition strategy enabled rapid elimination of homogeneous background regions, thereby reducing unnecessary computation while preserving diagnostically relevant cellular information. These characteristics make the framework potentially suitable for deployment in portable digital cytology systems and resource-constrained cervical cancer screening environments.

Despite these promising findings, several limitations remain. Although the proposed framework demonstrated competitive segmentation performance, it was not designed to outperform highly optimised deep learning architectures under severe overlap conditions. Cytoplasm segmentation remains particularly challenging in densely clustered cellular regions where boundaries become ambiguous even for advanced deep learning methods. In addition, the current evaluation was limited to image-level segmentation and did not include whole-slide image analysis or large-scale clinical deployment studies. Future work will focus on hybrid interpretable deep learning segmentation architectures, multiscale adaptive decomposition strategies, and deployment-oriented optimization for embedded microscopy and edge-based cytology systems. Overall, the findings demonstrate that adaptive hierarchical region decomposition remains a viable and computationally efficient approach for cervical cytology segmentation, particularly in deployment-oriented and low-resource healthcare environments. This technique will also be clinically validated on our developed low cost microscope (PapsAI).

## 5 Conclusion

Accurate nucleus and cytoplasm segmentation remains a fundamental requirement in automated Pap-smear analysis and cervical cancer screening systems. Reliable delineation of these cellular structures enables quantitative morphometric analysis, including assessment of nuclear size, shape, texture, and the clinically important nucleus-to-cytoplasm (N/C) ratio. However, robust segmentation remains challenging due to staining variability, intensity inhomogeneity, irregular morphology, and overlapping cellular structures. This study presented an Adaptive Quadtree-Based Segmentation framework for automated cervical cell segmentation using hierarchical region decomposition guided by adaptive statistical homogeneity analysis. The proposed framework integrates mean intensity, variance, entropy, local Otsu thresholding, adaptive region merging, overlap refinement, and morphological post-processing to improve discrimination between nucleus, cytoplasm, and background regions while maintaining computational efficiency and interpretability.

Experimental evaluation on both the Herlev dataset and the ISBI 2015 cervical cytology segmentation challenge dataset demonstrated that the proposed framework can produce competitive and reproducible segmentation results across both isolated and overlapping cervical cell environments. The framework achieved strong segmentation performance on the Herlev dataset with nucleus Dice coefficients exceeding 0.94 and ZSI values greater than 0.9034 across all diagnostic classes. Acceptable performance was also obtained on the more challenging ISBI 2015 overlapping-cell dataset, confirming the applicability of adaptive hierarchical decomposition beyond controlled single-cell scenarios.

Comparative benchmarking against U-Net and Attention U-Net showed that although deep learning architectures achieved superior performance under highly complex overlap conditions, the proposed framework remained competitive while requiring substantially lower computational resources and no iterative model training. Unlike deep learning approaches, the proposed method operates without GPU infrastructure, large annotated datasets, or extensive hyperparameter optimization, while maintaining interpretable and mathematically traceable segmentation behavior. The computational efficiency, transparency, and reduced hardware requirements make the framework particularly suitable for deployment in resource-constrained environments, portable digital cytology systems, and embedded microscopy platforms for decentralized cervical cancer screening. Overall, the proposed Adaptive Quadtree-Based Segmentation framework provides a practical, lightweight, and interpretable segmentation backbone for automated cervical cytology analysis and downstream computer-aided diagnosis systems.

## Data Availability

The original contributions presented in the study are included in the article/supplementary material, further inquiries can be directed to the corresponding author.
